# Anti-Neuroblastoma Activity of Gold Nanorods Bound with GD2 Monoclonal Antibody under Near-Infrared Laser Irradiation

**DOI:** 10.3390/cancers3010227

**Published:** 2011-01-06

**Authors:** Ching-An Peng, Chung-Hao Wang

**Affiliations:** 1 Department of Chemical Engineering, Michigan Technological University, 1400 Townsend Drive, Houghton, MI 49931, USA; 2 Department of Chemical Engineering, National Taiwan University, No. 1, Sec. 4, Roosevelt Rd., Taipei 10617, Taiwan

**Keywords:** gold nanorod, near-infrared laser, neuroblastoma, disialoganglioside, monoclonal antibody, thiolated chitosan, photothermolysis

## Abstract

High-risk neuroblastoma is one of the most common deaths in pediatric oncology. Current treatment of this disease involves a coordinated sequence of chemotherapy, surgery, and radiation. Further advances in therapy will require the targeting of tumor cells in a more selective and efficient way so that survival can be improved without substantially increasing toxicity. To achieve tumor-selective delivery, disialoganglioside (GD2) expressed by almost all neuroblastoma tumors represents a potential molecular target that can be exploited for tumor-selective delivery. In this study, GD2 monoclonal antibody (anti-GD2) was conjugated to gold nanorods (GNRs) which are one of anisotropic nanomaterials that can absorb near-infrared (NIR) laser light and convert it to energy for photothermolysis of tumor cells. Thiolated chitosan, due to its biocompatibility, was used to replace cetyltrimethylammonium bromide (CTAB) originally used in the synthesis of gold nanorods. In order to specifically target GD2 overexpressed on the surface of neuroblastoma stNB-V1 cells, anti-GD2 was conjugated to chitosan modified GNRs (CGNRs). To examine the fate of CGNRs conjugated with anti-GD2 after incubation with neuroblastoma cells, rhadoamine B was labeled on CGNRs functionalized with anti-GD2. Our results illustrated that anti-GD2-conjugated CGNRs were extensively endocytosed by GD2^+^ stNB-V1 neuroblastoma cells via antibody-mediated endocytosis. In addition, we showed that anti-GD2 bound CGNRs were not internalized by GD2^−^ SH-SY5Y neuroblastoma cells. After anti-GD2-linked CGNRs were incubated with neuroblatoma cells for six hours, the treated cells were further irradiated with 808 nm NIR laser. Post-NIR laser exposure, when examined by calcein-AM dye, stNB-V1 cells all underwent necrosis, while non-GD2 expressing SH-SY5Y cells all remained viable. Based on the *in vitro* study, CGNRs bound with anti-GD2 has the potential to be utilized as a therapeutic thermal coupling agent that generates heat sufficient to selectively kill neuroblastoma cells under NIR laser light exposure.

## 1. Introduction

Neuroblastoma is a solid tumor cancer that originates in the nerve tissue of the neck, chest, abdomen or pelvis, but most commonly in the adrenal gland. High-risk neuroblastoma (*i.e.*, stage IV) is one of the most devastating diagnoses a child can receive. In spite of aggressive treatment with surgery, chemotherapy and radiation, the overall long-term survival rate of patients with advanced stage neuroblastoma has only marginally prolonged [1]. This dismal prognosis underscores the necessity in the exploration of alternative therapies. Of potential approaches developed to overcome such treatment failures, targeting disialoganglioside (GD2) antigen is a promising one because GD2 is widely expressed by neuroblastoma, while its expression in normal tissues such as cerebellum and peripheral nerves is at very low levels [2]. Anti-GD2 monoclonal antibodies with high affinity and specificity to neuroblastoma cells have been used in clinical trials to kill malignant cells through both complement and cell-mediated lysis [3]. Liposomes tagged with anti-GD2 monoclonal antibody have been used to deliver anti-neoplastic agents to GD2^+^ neuroblastoma cells [4]. Epstein-Barr virus-specific T cells engineered to express GD2 antigen receptors have been shown to develop effective immune-based therapies for neuroblastoma [5]. Recently, *in vitro* photothermolysis of neuroblastoma cells by carbon nanotubes conjugated with anti-GD2 monoclonal antibody under near-infrared (NIR) laser light exposure has been demonstrated [6]. Although anti-GD2 bound carbon nanotubes (CNTs) could be internalized into neuroblastoma cells and CNT-laden neuroblastoma cells were destroyed using 808-nm NIR irradiation, the potential safety concern of using CNTs for further clinical studies remains. Since gold colloids have a long history known for their aesthetic appeal and therapeutic properties [7], gold nanorods (GNRs) reported as potential photothermal nanoabsorbers are therefore selected for this study.

Interest in rod-shaped gold nanoparticles arises from the photophysical properties of these anisotropic nanoscale-sized materials. The GNRs exhibit both transverse and longitudinal plasmon bands. The former one is located in the visible region peaked around 520 nm. The position of the latter one can be confined in the near-infrared region by tuning the aspect ratio of GNRs. Because of their unique plasmonic properties, applications of GNRs have been documented in gene delivery [8], chemical sensing [9], medical diagnostics [10], and photothermal destruction of pathogenic bacteria [11]. For cancer therapy, thanks to NIR absorption feature of GNRs, optical excitation with NIR light wavelength can penetrate tissues with minimal attenuation and selectively ablate GNR-targeted cancer cells by localized hyperthermia [12]. Due to its simplicity and robustness, seed-mediated growth method has been widely utilized for the synthesis of GNRs [13,14]. The wet chemical synthetic routes consist of (i) using a strong reducing agent (sodium borohydride) to prepare gold seed nanoparticles from gold salt (tetrachloroaurate), (ii) utilizing a weak reducing agent (ascorbic acid) to reduce more gold salt onto the gold seed particles, and then (iii) harnessing a structure-directing surfactant (cetyltrimethylammonium bromide - CTAB) to facilitate the formation of rod shapes. In order to obtain finer control of nanorod's aspect ratio (length/width ratio) and high yield of rod-shaped nanoparticles, silver ion (silver nitrate) is used to facilitate the seed-mediated growth method.

**Figure 1.**
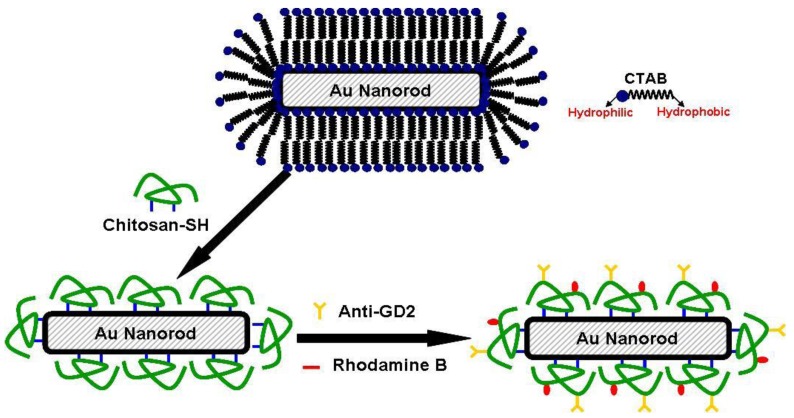
Schematic drawing of a thiolated chitosan modified gold nanorod (CGNR) conjugated with functional moieties. Thiolated chitosan was used to replace CTAB via robust Au-S bonds. CGNRs were further grafted with anti-GD2 for specific cell targeting and labeled with rhodamine B for fluorescent detection of CGNRs.

Since the seed-mediated growth method utilizes CTAB as the surfactant for the preparation of GNRs, large amount of CTAB dispersed in aqueous solution could lead to high cytotoxicity. Studies have shown that cytotoxicity of CTAB-passivated GNRs can be reduced by ligand exchange with phosphatidylcholine [15] and thiolated polyethylene glycol [16]. Polyelectrolyte encapsulation of CTAB-stabilized GNRs has also been reported as an approach to mitigate the cytotoxicity issue [17]. In the present study, low-molecular-weight water-soluble chitosan, due to its good biocompatibility, was covalently grafted with thiol groups. The synthesized thiolated chitosan was employed to replace CTAB originally used to stabilize the suspension of GNRs created by the seed-mediated growth method. The schematic illustration is shown in Figure 1.

GNRs stabilized by thiolated chitosan (CGNRs) were further functionalized with GD2 monoclonal antibody in order to specifically target neuroblastoma cells which express abundant GD2 on the cell surface. The specific binding of anti-GD2 functionalized CGNRs (anti-GD2-CGNRs) against GD2^+^ neuroblastoma cells *in vitro* and the ensuing ingestion of fluorescent labeled anti-GD2-CGNRs were investigated. After anti-GD2-CGNRs were specifically targeted to GD2^+^ neuroblastoma cells and then endocytosed by the cells, an 808-nm NIR laser with appropriate intensity and irradiation time was harnessed to excite the thermal absorber CGNR, and thereby resulted in photothermal ablation of neuroblastoma cells.

## 2. Results and Discussion

### 2.1. Characterization of GNRs and CGNRs

**Figure 2.**
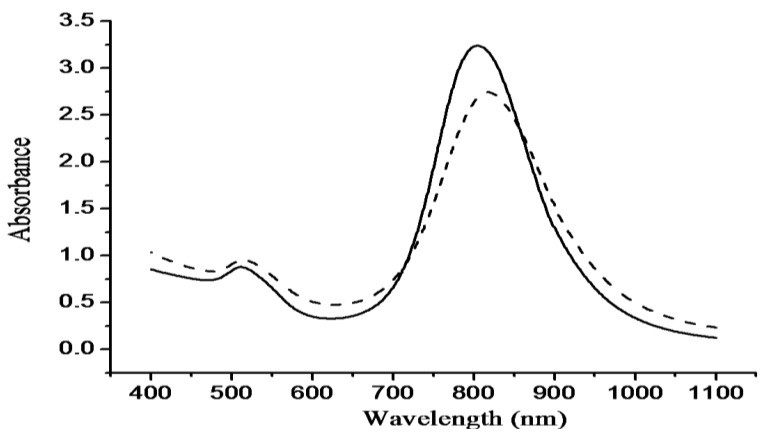
Absorption spectra of GNRs (solid line) and CGNRs (dashed line).

**Figure 3.**
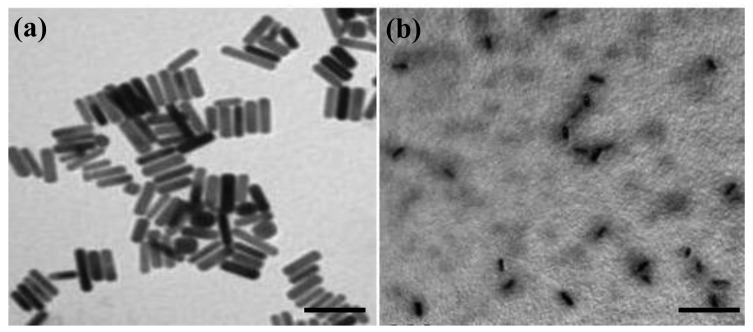
TEM images of (**a**) GNRs with an average aspect ratio of 3.9 (scale bar = 100 nm) and (**b**) CGNRs (scale bar = 200 nm) surrounded with a grey shell of thiolated chitosan.

The optical absorption spectra shown in Figure 2 without significant change in transverse and longitudinal plasmon bands indicated thiolated chitosan replacing CTAB on GNRs by ligand exchange can maintain stable CGNR suspension and sustain the optical property of GNRs. A TEM image of GNRs is shown in Figure 3(a), with an aspect ratio around 3.9 that can result in intense longitudinal absorption peaked in the vicinity of 800 nm which overlaps the region of minimum photon absorption by human tissues. In the TEM micrograph shown in Figure 3(b) for CGNRs, there is a gray shell encompassed around each GNR, suggesting GNRs were capped with thiolated chitosan via Au-S binding. The size distributions of GNRs and CGNRs were determined by dynamic light scattering with average size of 66 nm and 84.9 nm, respectively. Since CTAB and chitosan are positively charged materials, zeta potentials of GNRs and CGNRs were measured to be 28.7 and 26.6 mV, respectively.

**Figure 4.**
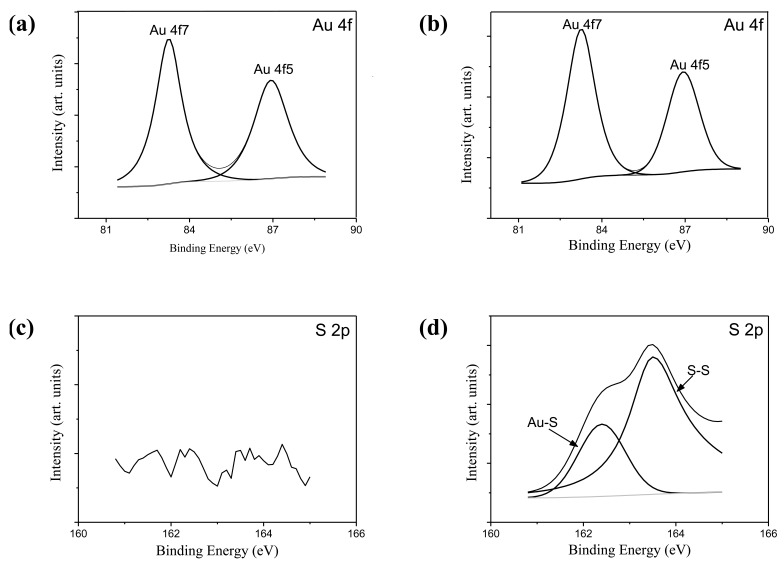
XPS spectra of GNRs and CGNRs. Au4f spectra of (**a**) GNRs and (**b**) CGNRs; S2p spectra of (**c**) GNRs and (**d**) CGNRs.

Figure 4(a) and (b) show Au4f spectra of GNRs and CGNRs, respectively. There was no difference between these two spectra; indicating the composition of Au was not altered by the ligand exchange process. No Au-S binding energy was detected in the S2p spectrum of GNRs shown in Figure 4(c). Conversely, two prominent binding energy doublet peaks were determined from the S2p spectrum of CGNRs given in Figure 4(d). The weak spectrum with binding energy peaking at 162.4 eV was attributed to Au-S bond. While, the other spectrum peak at 163.5 eV could result from the X-ray radiation damage to the sample, causing progressive Au-S bond breaking and the creation of new sulfur species [18]. It is clearly demonstrated from Figure 4 that thiolated chitosan can be used to replace large amounts of CTAB and maintain stabilized GNRs via Au-S binding. A significant reduction of CTAB bromide signal of CGNRs revealed only residual amounts of CTAB molecules left on CGNRs after the ligand exchange process (data not shown).

### 2.2. Cell-GNRs Interaction

**Figure 5.**
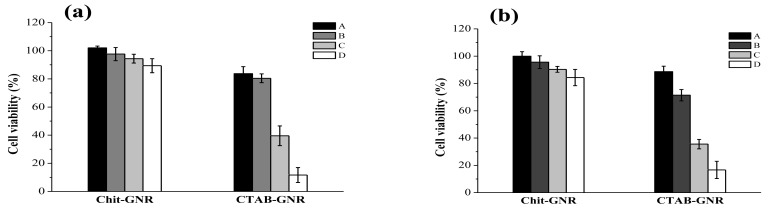
Cell viability of (**a**) SH-SY5Y and (**b**) stNB-V1 cells after treatment with different concentrations of GNRs and CGNRs for 24 h as determined by the MTT assay. The bar labels A to D stand respectively for 0.025, 0.05, 0.1, and 0.2 mM (as Au atoms) of twice-centrifuged CGNRs and GNRs in the culture media. Data shown here are the mean ± SD of triplicate experiments.

As shown in Figure 5, after stNB-V1 and SH-SY5Y cells were challenged with GNRs and CGNRs concentrations ranging from 0.025 to 0.2 mM for 24 h, cell viability decreased significantly along with the increment of GNR concentration. For example, GNRs with a concentration of 0.1 mM caused viability of SH-SY5Y and stNB-V1 cells to drop to 39% and 36%, respectively. However, these two cell types remained around 90% viable up to the highest CGNR concentration employed for this study. This indicates that decreasing the CTAB concentration in GNR suspension by centrifugation twice would not render GNRs biocompatible. While, thiolated chitosan used to replace CTAB from GNRs could alleviate cytotoxicity to a large extent.

**Figure 6.**
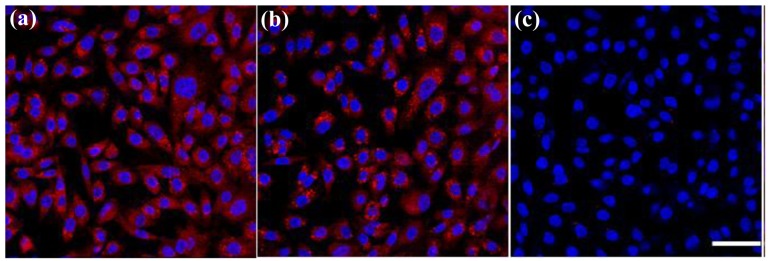
Fluorescent images of stNB-V1 cells pre-treated separately with (**a**) 0.05, (**b**) 0.5, (**c**) 5 μg of free anti-GD2 monocolonal antibody for 12 h and then challenged with 0.1 mM anti-GD2-CGNRs labeled with rhodamine B for 6 h (scale bar = 25 μm). The blue fluorescence is DAPI used to stain the cell nucleus and the red fluorescence is rhodamine B used to label CGNRs.

**Figure 7.**
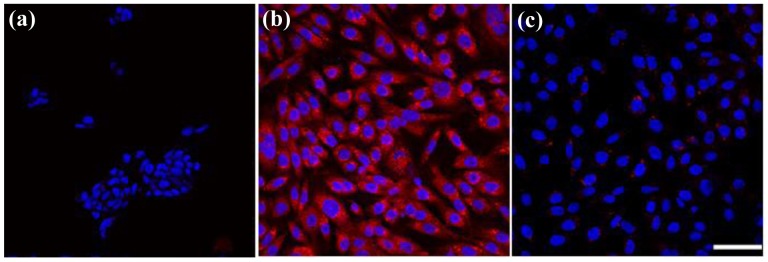
Fluorescent images of (**a**) SH-SY5Y and (**b**) stNB-V1 cells treated with rhodamine B tagged anti-GD2-CGNRs for 6 h, and (**c**) stNB-V1 treated with CGNRs only tagged with rhodamine B for 6 h (scale bar = 25 μm). The blue fluorescence is DAPI used to stain the cell nucleus and the red fluorescence is rhodamine B used to label CGNRs.

In competition experiments (shown in Figure 6), we observed that the binding of anti-GD2-CGNRs to GD2-expressing stNB-V1 neuroblastoma cells was completely abolished by the presence of free anti-GD2 in excess (Figure 6(c)). These results indicate that the enhanced association of GD2-targeted CGNRs to neuroblastoma cells is indeed due to antibody-mediated specific recognition of GD2 antigens overexpressed on neuroblastoma cells. Furthermore, antibody-mediated endocytosis (as shown in Figure 7(b)) was not observed when stNB-V1 cells were challenged by CGNRs labeled only with rhodamine B for 6 h (Figure 7(c)) because CGNRs without anti-GD2 conjugation could not be specifically recognized by stNB-V1 cells. As shown in Figure 7(c), sparkling red fluorescence of rhodamine B labeled CGNRs was detected in the cytosol of stNB-V1 cells. This is because CGNRs labeled with rhodamine B were internalized by stNB-V1 cells via non-specific surface binding. Moreover, SH-SY5Y cells lacking GD2 expression on the cell surface did not ingest anti-GD2-CGNRs after 6 h treatment (Figure 7(a)). Taken together, our results demonstrate that CGNRs bound with anti-GD2 antibody could specifically target GD2 expressing neuroblastoma cells but not the cells without GD2 expression. In addition, the specific targeting of anti-GD2-CGNRs to neuroblastoma cells is by virtue of antigen-antibody recognition.

### 2.3. Photothermolysis with NIR Laser Irradiation

**Figure 8.**
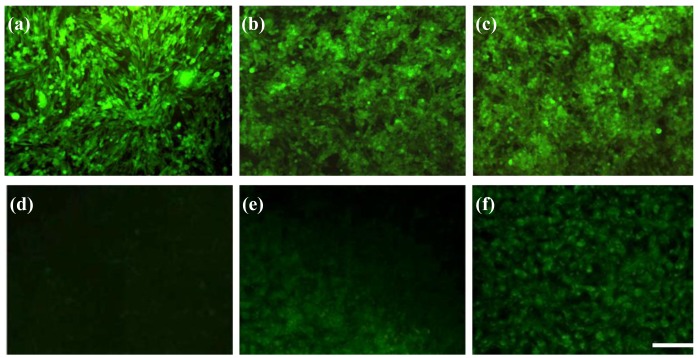
Photothermal treatment of SH-SY5Y (**a-c**) and stNB-V1 (**d-f**) cells. After SH-SY5Y and stNB-V1 were incubated with anti-GD2-CGNRs for 6 h, 808 nm NIR laser was harnessed to beam the cells from 0.2 to 2 W/cm^2^ within 10 min and then maintaining at 2 W/cm^2^ for an additional 5 min. After staining with 2.5 μM calcein-AM dye, fluorescent images of cells were taken (**a, d**) within, (**b, e**) on the edge of, and (**c, f**) outside the NIR laser-shining zone (scale bar = 100 μm). Green fluorescence indicates viable cells, in contrast to dead cells which reveal no fluorescence.

Photothermal treatment was first carried out separately for GD2^+^ stNB-V1 and GD2^−^ SH-SY5Y cells 6h post-incubation with 0.1 mM anti-GD2-CGNRs. After staining with calcein-AM dye, 496 nm blue light was used to illuminate cells. In this way, if a cell is viable, it emits green fluorescence, whereas a dead cell will not emit any fluorescent light. After incubation with anti-GD2-CGNRs for 6 h, the fluorescent images of SH-SY5Y cells located in the NIR laser-beaming zone, on the laser-shining edge and far from the beam zone were taken and are shown in Figure 8(a)–(c), respectively. Apparently, the GD2^−^ cells located in all of the three zones remained viable, as indicated by their green fluorescence. However, for GD2^+^ cells treated with anti-GD2-CGNRs for 6 h and then irradiated with NIR laser light, the cells located in the laser-shining zone (Figure 8(d)) did not reveal green fluorescence (*i.e.*, cell necrosis), compared with the cells harbored far away from the beam zone (Figure 8(f)) yielding green fluorescence indicating cell viability. Figure 8(e) represents the cells located on the edge of the NIR laser irradiation, clearly illustrating the boundary between necrotic (dark) and viable cells (green).

**Figure 9.**
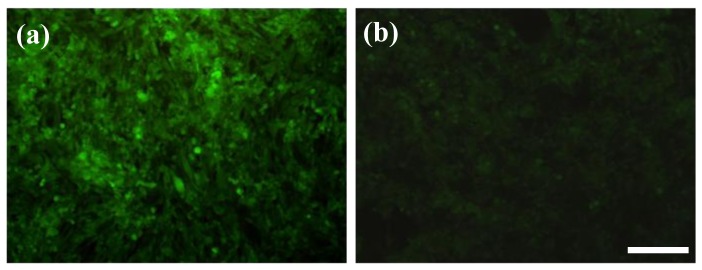
Photothermal treatment of SH-SY5Y and stNB-V1 cells mixed with the ratio of (**a**) 4:1 and (**b**) 1:4. Mixed cell population was co-cultured with 0.1 mM anti-GD2-CGNRs for 6 h, beamed with 808 nm NIR laser light with intensity from 0.2 to 2 W/cm^2^ within 10 min and then maintaining at 2 W/cm^2^ for an additional 5 min. After stained with 2.5 μM calcein-AM dye, fluorescent images of cells were taken within the NIR laser-shining region (scale bar = 100 μm).

To clarify the feasibility of selectively eradicating GD2^+^ cells by anti-GD2-CGNRs, the mixtures of GD2^−^ and GD2^+^ cells with 4:1 and 1:4 ratios were separately challenged with 0.1 mM anti-GD2-CGNRs, irradiated with NIR laser light, stained with calcein AM dye, and then cell images were taken by a fluorescent microscope. Figure 9(a) shows that the mixed population with higher amounts of GD2^−^ cells (*i.e.*, 4:1) was able to yield intense fluorescence, which was consistent with much more viable GD2^−^ cells in the mixed population; while the mixture population with higher amounts of GD2^+^ cells (*i.e.*, 1:4) gave weak fluorescent intensity of calcein-AM due to less viable GD2^−^ cells in the population (shown in Figure 9(b)).

Althoug the *in vitro* studies demonstrated anti-GD2-CGNR-mediated photothermolyisis with NIR laser irradiation is a promising approach to selectively destroy GD2^+^ neuroblastoma cells, its potential use as a therapeutic modality for neuroblastoma remains to be validated by animal models. In fact, the major limitation of NIR laser treatment for neuroblastoma is that most neuroblastomas are deep-seated in the retroperitoneum or mediastinum where NIR light can hardly reach. To circumvent the penetration depth limitation, NIR laser irradation probably needs to be harnessed right after surgical removal of neuroblastoma. After resection of primary neuroblastoma, it is difficult to surgically clean up residual neuroblastoma cells, which are likely to cause tumor relapse leading to neuroblastoma treatment failures. Hence, we surmise that anti-GD2-CGNR-mediated photothermolyisis induced by NIR laser could provide a therapeutic adjunct for the eradiation of refractory residual neuroblastoma cells. The rationale is that gold nanorods conjugated with anti-GD2 can be sprinkled on residual neuroblastoma cells in the post-resection tumor bed and act as thermal nanoscalpels when exposed with NIR laser light. After such an adjunct treatment, conventional therapies including focal radiotherapy and high-dose chemotherapy with autologous hematopoietic stem cells should be conducted accordingly to prolong disease stabilization.

## 3. Experimental Section

### 3.1. Materials

Tetrachloroauric acid (HAuCl_4_), cetyltrimethylammonium bromide (CTAB), sodium borohydride (NaBH_4_), ascorbic acid, silver nitrate (AgNO_3_), thioglycolic acid (TGA), chitosan (MW: 100∼300 kDa, degree of deacetylation 80%), trypsin, 1-ethyl-3-(3-dimethylaminopropyl) carbodiimide hydrochloride (EDC), 2-(N-morpholino)ethanesulfonic acid (MES), 4′-6-diamidino-2-phenylindole (DAPI), rhodamine B, methylthiazol tetrazolium (MTT), dimethyl sulfoxide (DMSO), and phosphotungstic acid were all purchased from Sigma-Aldrich (St. Louis, MO, U.S.). Calcein AM was purchased from Invitrogen (Carlsbad, CA, U.S.). Dulbecco's modified Eagle's medium/F12 (DMEM/F12) and fetal bovine serum (FBS) were purchased from HyClone (Logan, UT, U.S.). Mouse anti-human disialoganglioside GD2 monoclonal antibody (Clone 14.G2a) was purchased from Chemicon International, Inc (Temecula, CA, U.S.).

### 3.2. Preparation of Gold Nanorods

First, the gold seed particles were prepared by adding 1 mL 0.5 mM HAuCl_4_ to 1 mL 0.2 M CTAB solution. Then, 0.12 mL 0.01 M ice-cold NaBH_4_ was added with gentle mixing, which resulted in the formation of brownish yellow solution. Second, the gold nanorod growth solution was prepared by adding 5 mL 1 mM HAuCl_4_ to 5 mL 0.2 M CTAB solution. Then, 0.26 mL 4 mM AgNO_3_ and 67 μL 7.9 mM ascorbic acid were added. The solution color changed from dark yellow to colorless, while adding the ascorbic acid. Finally, 12 μL gold seed solution was added to the above solution. In order to remove the excessive CTAB, the gold nanorod solution was kept in a refrigerator and followed by centrifugation at 3000 rpm for 10 min.

### 3.3. Depolymerization of Chitosan

One gram of chitosan was completely dissolved in 100 mL 2% acetic acid solution, and then 4.25 mL of 35% H_2_O_2_ aqueous solution was added. The solution was stirred and reacted at 80 °C for 1 h. The reaction mixture thus obtained was filtered by a sintered funnel, and the filtrate was dialyzed (Cellu/Sep^®^ dialysis membrane, 3.5 kD cutoff, Membrane Filtration Products, Inc., Seguin, TX, U.S.) against deionized water. Then, the filtrate was dialyzed (Cellu/Sep^®^ dialysis membrane, 6-8 kD cutoff) extensively against deionized water, and the dialysate containing chitosan with MW ranging from 3.5 to 6 kDa. Low molecular-weight water-soluble chitosan was freeze-dried and collected in a powder form.

### 3.4. Synthesis of Thiolated Chitosan

Depolymerized chitosan dissolved into deionized water was reacted with TGA in the presence of EDC dissolved in 0.1 M MES buffer (pH = 5.5) for 12 h at room temperature. The molar ratio of chitosan/EDC/TGA was 1:5:10. To eliminate the unbound TGA and to isolate the chitosan conjugate, the reaction mixture was dialyzed (Cellu/Sep^®^ dialysis membrane, 3.5 kD cutoff) against deionized water containing 1% NaCl to reduce ionic interactions between the cationic chitosan and the anionic sulfhydryl TGA. The solution containing thiolated chitosan remaining in the dialysis tube was freeze-dried and stored for later use.

### 3.5. Preparation of Gold Nanorods Stabilized by Thiolated Chitosan

Two milliliters of 0.4 mM gold nanorods solution and 8 mL of 1 mM thiolated chitosan solution were mixed at room temperature for 2 days. The solution was centrifuged four times at 8,000 × g (Allegra X-22 centrifuge, F1010 rotor, Beckman Coulter, Fullerton, CA) for 15 min to remove excess thiolated chitosan and obtain CGNRs.

### 3.6. Characterization of GNR and CGNR

Absorption spectra of GNR and CGNR were obtained by a UV-Vis-NIR spectrometer (V-570, Jasco, Tokyo, Japan). The size distribution and zeta potential of GNR and CGNR were determined by a dynamic light scattering device (Zetasizer Nano-ZS, Malvern Instruments, Worcestershire, U.K.). TEM specimen was made by evaporating one drop of GNR (or CGNR) solution on a carbon-coated copper grid. TEM micrographs were taken by transmission electron microscope (JEM-1230, JOEL, Tokyo, Japan) operating at 100 kV. Aqueous solution of 1% phosphotungstic acid was used as the negative stain reagent. The compositions of CTAB stabilized gold nanorods (GNRs) and thiolated chitosan modified gold nanorods (CGNRs) were analyzed by X-ray photoelectron spectroscopy (XPS, Theta Probe, Thermo Scientific, U.K.). A monochromatic Al Kα X-ray source at 1486.68 eV was used. The X-ray power, the pass energy of the analyzer and the take-off angle of the photoelectron were set at 100 W, 20 eV, and 50°, respectively. The energy resolution of this setup was about 0.5 eV, estimated by the Ag 3d5/2 peak width at the measurement condition. C1s, Au4f, Br3d and S2p levels were recorded. The binding energy was calibrated using the C1s peak energy (284.8 eV) as an energy standard.

### 3.7. Cytotoxicity of GNRs and CGNRs

GD2^+^ stNB-V1 neuroblastoma cells (obtained from Dr. Christina Ling Chang, Institute of Molecular Medicine, National Cheng-Kung University, Tainan, Taiwan) and GD2^−^ SH-SY5Y neuroblastoma cells (American Type Culture Collection, Manassas, VA) were cultured in DMEM/F12 medium supplemented with 10% FBS. To examine the biocompatibility of GNRs and CGNRs, 5 × 10^5^ stNB-V1 and SH-SY5Y cells per well were incubated separately in a 24-well culture plate and cultivated at 37 °C in humidified air containing 5% CO_2_. After 24-h incubation, cell culture media were replaced separately with various volumes (50, 100, 200, 400 μL) of 1 mM GNRs or CGNRs premixed with corresponding volumes (1.95, 1.9, 1.8, 1.6 mL) of fresh DMEM/F12 supplemented with 10% FBS. The final concentrations of GNRs or CGNRs in a 24-well plate were 0.025, 0.05, 0.1, 0.2 mM. After treatment with various amounts of GNRs or CGNRs for 24 h, 2 mL of the mixture of MTT assay reagent (4 g/L) and culture medium (volume ratio = 1:9) was added into each well to culture for 4 h. The mixture was removed and 2 mL of DMSO was added into each well for 20 min. Finally, 200 μL of DMSO solution was transferred from a 24-well plate into a 96-well plate and the absorption intensity was detected at 450 nm by a microplate reader (SpectraMax M2, Molecular Device, Sunnyvale, CA, U.S.).

### 3.8. Functionalization of CGNRs

Two hundred microliters of 0.2 M MES buffer solution (pH = 6.8) was added to CGNR solution under stirring. Then, 0.5 μg anti-GD2 activated by 19 μg (1 μL of 0.1 M) EDC was added into the MES buffer solution containing CGNRs. After 12 h reaction, the solution was centrifuged four times at 8,000 × g for 15 min to remove residual anti-GD2 and obtain CGNRs grafted with anti-GD2 (*i.e.*, anti-GD2-CGNRs). For labeling anti-GD2-CGNRs with rhodamine B, 200 μL of 0.2 M MES buffer solution (pH = 6.8) was added to anti-GD2-CGNR solution under stirring. Then, 1 μg rhodamine B activated by 19 μg (1 μL of 0.1 M) EDC was added to the MES buffer solution containing anti-GD2-CGNRs. After 12 h reaction, the solution was centrifuged four times at 8,000 × g to remove residual rhodamine B and obtain anti-GD2-CGNRs labeled with fluorescent rhodamine B.

### 3.9. Endocytosis of Functionalized CGNRs

SH-SY5Y and stNB-V1 cells (2 × 10^5^ cells/mL) were inoculated separately in T-25 flasks and cultivated for 24 h. Prior to the treatment of rhodamine B labeled anti-GD2-CGNRs, SH-SY5Y and stNB-V1 cells were separately detached from the T-25 flasks by trypsinization and 1 × 10^5^ cells/mL was loaded onto coverslip-bottomed Petri dishes (MatTek, Ashland, MA) containing 2 mL culture media. Then, 0.1 mM of rhodamine B labeled anti-GD2-CGNRs were added into the Petri dishes containing SH-SY5Y and stNB-V1 cells for 6 h incubation, respectively. After replacement with fresh culture media, 20 μg DAPI was added to each dish and allowed to incubate for 1 h at room temperature for nucleus staining. Cell images were taken by laser confocal microscopy (Leica TCS SP5, Wetzlar, Germany). As a negative control set, GD2^−^ SH-SY5Y cells were challenged with rhodamine B labeled anti-GD2-CGNRs for 6 h and then micrographic fluorescent images were obtained. For comparison, CGNRs (without anti-GD2 conjugation) labeled with only rhodamine B were also used to incubate with stNB-V1 cells for 6 h and fluorescent images of cells were taken.

To validate that endocytosis of anti-GD2-CGNRs is facilitated by specific antibody-mediated recognition, GD2^+^ stNB-V1 cells were pre-treated separately with 0.05, 0.5, and 5 μg of free anti-GD2 monoclonal antibody for 12 h to block GD2 antigens on the cell surface and then challenged with rhodamine B labeled anti-GD2-CGNRs for 6 h.

### 3.10. NIR-Mediated Photothermolysis

For the laser irradiation experiment, a continuous-wave fiber-coupled laser integrated unit with wavelength 808 nm (Opto Power Corp., Tucson, AZ, U.S.) was used. Prior to NIR-mediated photothermolysis, stNB-V1 and SH-SY5Y were cultured separately in a 24-well tissue cultured plate for 24 h, and then treated with 0.1 mM anti-GD2-CGNRs for 6 h. The culture plate wells were washed with phosphate-buffered saline three times to remove any residual anti-GD2-CGNRs and then replaced with fresh culture media before the cells exposure to NIR laser light The intensity of NIR laser beam was tuned from 0.2 to 2 W/cm^2^ within 10 min and then maintained at 2 W/cm^2^ for an additional 5 min. The laser beam was delivered to the target through a 1.5 m long, 600 μm single core fiber with a numerical aperture of 0.37, followed by a 25 mm focal length fused-silica biconvex lens. The focused spot size was 1.2 cm. After NIR laser illumination, cells were stained with 2.5 μM calcein AM to examine viability under fluorescent microscopy. GD2^+^ stNB-V1 and GD2^−^ SH-SY5Y cells were further mixed in various ratios (1:4 and 4:1) and then challenged with 0.1 mM anti-GD2-CGNRs. After 6 h incubation and replacement with fresh medium, the mixed cell populations were exposed to 808 nm NIR laser with intensity tuned from 0.2 to 2 W/cm^2^ within 10 min and then maintained at 2 W/cm^2^ for an additional 5 min. Fluorescent images were taken after the treated cells were stained with calcein-AM for 1 h.

## 4. Conclusions

Our results demonstrated clearly that anti-GD2 labeled CGNRs could selectively target GD2^+^ cells from a mixture of GD2^+^ and GD2^−^ cells, releasing substantial heat in the nanoenvironment after exposure to NIR laser light, and thereby lead to thermoablation of GD2^+^ cells rather than GD2^−^ cells. This implies that specific targeting of GD2^+^ cells with CGNRs functionalized with anti-GD2 monoclonal antibody could selectively destroy GD2^+^ cells by NIR laser exposure without collateral damage to the surrounding healthy cells. In summary, our data suggest that chitosan modified gold nanorods combined with NIR laser-induced photothermolysis might be a promising anti-cancer modality to treat neuroblastomas that highly express GD2 antigen.
